# Slow freezing versus vitrification for the cryopreservation of zebrafish (*Danio rerio*) ovarian tissue

**DOI:** 10.1038/s41598-019-51696-7

**Published:** 2019-10-25

**Authors:** Lis S. Marques, Ana A. N. Fossati, Rômulo B. Rodrigues, Helen T. Da Rosa, Aryele P. Izaguirry, Juliana B. Ramalho, José C. F. Moreira, Francielli Weber Santos, Tiantian Zhang, Danilo P. Streit

**Affiliations:** 1 Pós-Graduação em Zootecnia, Departamento de Zootecnia, Universidade Federal do Rio Grande do Sul, Porto Alegre, RS Brazil; 2 Departamento de Bioquímica, Universidade Federal do Rio Grande do Sul, Porto Alegre, RS Brazil; 3 Laboratório de Biotecnologia da Reprodução (Biotech), Universidade Federal do Pampa, Uruguaiana, RS Brazil; 4 Faculty of Science and Technology, Bournemouth University, Dorset, UK

**Keywords:** Biophysical methods, Animal biotechnology, Assay systems, Systems biology

## Abstract

The aim of the present study was to compare the efficiency of vitrification and slow freezing techniques for the cryopreservation of zebrafish ovarian tissue containing immature follicles. In Experiment 1, assessment of cell membrane integrity by trypan blue exclusion staining was used to select the best cryoprotectant solution for each cryopreservation method. Primary growth (PG) oocytes showed the best percentage of membrane integrity (63.5 ± 2.99%) when SF4 solution (2 M methanol + 0.1 M trehalose + 10% egg yolk solution) was employed. The vitrification solution, which presented the highest membrane integrity (V2; 1.5 M methanol + 5.5 M Me_2_SO + 0.5 M sucrose + 10% egg yolk solution) was selected for Experiment 2. Experiment 2 aimed to compare the vitrification and slow freezing techniques in the following parameters: morphology, oxidative stress, mitochondrial activity, and DNA damage. Frozen ovarian tissue showed higher ROS levels and lower mitochondrial activity than vitrified ovarian tissue. Ultrastructural observations of frozen PG oocytes showed rupture of the plasma membrane, loss of intracellular contents and a large number of damaged mitochondria, while vitrified PG oocytes had intact mitochondria and cell plasma membranes. We conclude that vitrification may be more effective than slow freezing for the cryopreservation of zebrafish ovarian tissue.

## Introduction

Currently, the most widely used fish model is zebrafish (*Danio rerio*), which presents 12,719 genes in common with humans^1^, and shows high fertility^2^ and embryonic transparency^3^. Due to the use of the species as an animal model for scientific research, numerous specific strains and lines have been created and are stored as live animals in resource centers worldwide, such as the Zebrafish International Resource Center (University of Oregon, Eugene, OR). The preservation of the genetic resources of this important model and other valuable fish is a challenge that requires attention, and the cryopreservation of gametes, embryos and gonads is a useful approach to address these challenges.

The cryopreservation of fish oocytes or embryos has not been successful due to the large size, high intracytoplasmic lipid content^4^ and membrane impermeability^5^ of these cells. However, the cryopreservation of fish sperm was first reported more than 50 years ago^6^, and since then, sperm cryopreservation protocols have been developed in more than 200 species of fish^7^. However, most research has focused on large species, such as salmonids and carp, and few studies have addressed aquarium fish^8^. The factors that hamper studies on sperm cryopreservation in aquarium fish, such as zebrafish, include the small size of the animal and the limited semen availability, where 3.3 μL of semen is typically obtained in a single collection^9^. Consequently, sperm cryopreservation in these species presents challenges, such as experimental design, gamete collection and artificial fertilization, especially when using live fish. Ovarian tissue cryopreservation was first proposed in zebrafish by Zampolla *et al*.^10^. These authors suggested that ovarian fragments containing follicles in stages I and II (immature) were less susceptible to cryoinjury than ovarian follicles in stage III (vitellogenic). Immature follicles have a smaller size, which results in a higher surface/volume ratio; hence, these tissues are more permeable to water and solutes, increasing the chances of survival during cryopreservation. The efficiency of the vitrification technique in the cryopreservation of zebrafish ovarian tissue was investigated in 2013, where authors reported a high loss of follicular viability after warming^11^. However, this study only used fragments of ovarian tissue containing stage III follicles (vitellogenic). In a recent study, we obtained a high viability of immature follicles (76% in stages I and 43% in stages II) following zebrafish ovarian tissue vitrification^12^. These data show that immature follicles are more likely to survive after vitrification than follicles at more advanced stages of maturation (III, IV and V). The same vitrification protocol was applied to *Piaractus mesopotamicus* and the immature follicles showed 70% of viability using histological analysis and two staining protocols (trypan blue and fluorescein diacetate combined with propidium iodide)^13^.

The two most commonly used cryopreservation methods for the preservation of animal germplasm are slow freezing and vitrification. Both methods are based on the same principles of protecting cells from damage due to intracellular ice formation, excessive cell dehydration and changes in solute concentrations. Slow freezing requires a controlled and gradual temperature procedure; however, there is the risk of crystallization that is correlated with membrane damage. On the other hand, highly concentrated cryoprotective solutions in vitrification, enables the sudden reduction of temperature, allowing the passage from a liquid to an amorphous vitreous state, avoiding the formation of ice crystals. However, the absence of crystallization does not exclude the possibility of cellular damage, such as oxidative stress and DNA fragmentation. Both cooling and heating procedures increase the production of reactive oxygen species (ROS) causing changes in oxidative metabolism. Thus, to evaluate oocyte quality after cryopreservation of ovarian tissue, the following assessments were performed: membrane integrity, oocyte morphology, DNA damage and oxidative stress parameters (levels of reactive oxygen species and antioxidant capacity). To date, no study has compared vitrification and slow freezing techniques in the cryopreservation of zebrafish ovarian tissue fragments. Therefore, the major aim of the present study was to compare the efficiency of vitrification and slow freezing techniques in the cryopreservation of zebrafish ovarian tissue containing immature follicles.

## Materials and Methods

### Experimental design

In Experiment 1, eight cryoprotectant solutions were tested in slow freezing, and four vitrification solutions were tested in the vitrification technique. Five replicates were performed for each group (cryopreserved treatments and control/fresh ovarian tissue).

In Experiment 2, ovarian tissue fragments were distributed among treatments (slow freezing, vitrification, and control/fresh ovarian tissue). Five replicates were performed in the histology analysis and mitochondrial activity assay, and eight replicates were performed in the biochemical analysis and comet assay.

The animal experiment reported in the present study was conducted in accordance with the Conselho Nacional de Controle e Experimentação Animal - CONCEA (National Council for Control and Animal Experimentation) and approved by the Ethics Committee of the Federal University of Rio Grande do Sul. Project number: 29303.

### Fish care and ovarian fragment collection

Eighteen-week-old females with 0.36 ± 0.07 g average weight were euthanized with a lethal dose of tricaine methane sulfonate (0.6 mg/mL, pH 7.4), followed by decapitation. The ovaries were immediately collected after decapitation and placed in 90% Leibovitz L-15 medium (pH 9.0). The body and ovaries were weighed (average 0.03 ± 0.01 g), and the average gonadosomatic index (GSI = [ovaries weight/body weight] × 100) was 7.20 ± 2.44. Fragments containing primary growth (PG) oocytes were carefully dissected from the ovaries and cut into thin slices (2 mm) using syringe needles.

Two or three fragments were collected from each female and randomly distributed among treatments.

## Experiment 1

The aim of Experiment 1 was to select the best cryoprotectant solution according to the results of membrane integrity assay for each cryopreservation method (slow freezing and vitrification). The solution that presented the best result, in each method, was used in Experiment 2.

### Cryoprotectant solutions

The composition and concentrations of the cryoprotectant solutions for slow freezing were elaborated according to the results obtained in previous studies with zebrafish follicles^14,15^, as well as the data reported by Lee *et al*.^16^ on the slow freezing of rainbow trout (*Oncorhynchus mykiss*) testicles using Bicell recipient (Nihon Freezer Co., Ltd., Tokyo, Japan).

In a previous study, where zebrafish ovarian tissue was vitrified, the solution comprised 1.5 M methanol + 5.5 M Me_2_SO + 0.5 M sucrose resulted in a 64% viability of early-stage follicles^12^. Thus, this cryoprotectant solution was used to verify the effect of substituting sucrose for trehalose and to evaluate the effects of supplementation with 10% egg yolk solution.

The egg yolk solution was prepared as described by Isachenko and Nayudu^17^, with some modifications. Fresh egg yolk was mixed with 90% L-15 medium (pH 9.0) at a 1:2 ratio and then centrifuged for 30 min at 10,000 rpm at 15 °C. Only the supernatant was used in the cryoprotectant solution. All cryoprotectant solutions tested in the present study are presented in Table 1.

**Table 1:** The composition of cryoprotectant solutions tested in vitrification (V) or slow freezing (SF).

CryoprotectantSolution	Methanol (M)	Me_2_SO (M)	Sucrose(M)	Trehalose (M)	Yolk solution (%)
V1	1.5	5.5	0.5		
V2	1.5	5.5	0.5		10
V3	1.5	5.5		0.5	
V4	1.5	5.5		0.5	10
SF1	2		0.1		
SF2	2		0.1		10
SF3	2			0.1	
SF4	2			0.1	10
SF5		2	0.1		
SF6		2	0.1		10
SF7		2		0.1	
SF8		2		0.1	10

V = cryoprotectant solution tested in the vitrification procedure. SF = cryoprotectant solution tested in the slow freezing procedure. Me_2_SO = dimethyl sulfoxide.

### Slow freezing

Ovarian tissue fragments were transferred to 1.2-mL cryotubes containing 500 µL of cryoprotectant solution at 4 °C. Samples were incubated in cryomedium at 4 °C for 60 min and then placed in a Bicell plastic freezing container (Nihon Freezer, Japan) and subsequently stored in a −80 °C freezer for 90 min, enabling a −1 °C/min cooling curve before immersion in liquid nitrogen.

After seven days, the cryotubes were removed from the liquid nitrogen for warming procedure. The cryotubes were thawed in water bath for 30 s at 28 °C, and then the samples were washed three times in 90% L-15 medium (pH 9.0, 22 °C) to remove cryoprotectants.

### Vitrification

Ovarian tissue fragments were transferred to 1.2-mL cryotubes containing 500 µL of equilibrium solution (1.5 M methanol and 2.75 M Me_2_SO) for 15 min at room temperature (22 °C ± 1 °C). Next, the equilibrium solution was removed, and 500 µL of vitrification solution (VS) was added. After 90 sec, VS was removed, and a minimum volume of medium (~10 µL) was left in the cryotubes. Then, the bottom of the cryotubes were placed in contact with liquid nitrogen, sealed and immediately plunged in liquid nitrogen. After seven days, the cryotubes were removed from the liquid nitrogen and thawed in water bath for 30 s at 28 °C. Subsequently, the ovarian tissue fragments were exposed to three warming solutions at 22 °C: the first warming solution, containing 1 M sucrose, for 1 min, followed by a second solution containing 0.5 M sucrose for 3 min and finally a third solution of 0.25 M sucrose for 5 min. The samples were washed three times in 90% L-15 medium (pH 9.0, 22 °C).

### Membrane integrity assay

Immediately after warming, cryopreserved and control follicles were isolated by gentle pipetting in Leibovitz L-15 medium. PG oocyteswere incubated in 0.4% trypanblue for 3 min and then washed three times in 90% L-15 medium. At least 100 PG oocytes in each group (cryopreserved treatments and fresh ovarian tissue fragments) were observedunder a light microscope.The unstained oocytes were considered membrane-intact oocytes (percentage of membrane integrity of PG oocytes), while the blue stained oocytes were considered membrane-damaged oocytes.

## Experiment 2

The aim of Experiment 2 was to compare the vitrification and slow freezing techniques based on the following parameters: morphology, oxidative stress, mitochondrial activity, and DNA damage.

Slow freezing and vitrification procedures and the methodology for obtaining ovarian tissue fragments were the same as those described in Experiment 1. V2 (1.5 M methanol + 5.5 M Me_2_SO + 0.5 M sucrose + 10% egg yolk solution) and SF4 (2 M methanol + 0.1 M trehalose + 10% egg yolk solution) cryoprotectant solutions were selected to be used in Experiment 2.

### Histological analysis

Ovarian tissue fragments were fixed in 10% buffered formalin (pH 7.2–7.4) for 24 h, then embedded in paraffin wax, sliced (5 µm), and stained with hematoxylin and eosin (HE). Microscopic evaluation was performed to assess the morphological integrity of PG oocytes following both cryopreservation procedures. In each group (vitrification, slow freezing and control/fresh ovarian tissue fragments), at least 100 PG oocytes with a visible nucleus were counted and categorized as intact and non-intact (percentage of cell integrity). Non-intact oocytes were those with presence of pyknotic nuclei (nucleus damage; condensation of the chromatin and shrinkage of the nucleus) and/or follicular membrane rupture (membrane damage).

### Ultrastructural analysis by transmission electron microscopy (TEM)

Fresh, vitrified and frozen ovarian tissue fragments were fixed in 2.5% glutaraldehyde +2% paraformaldehyde in 0.1 M phosphate buffer (pH 7.2–7.4) for 24 h. After osmium tetroxide post fixation and alcohol dehydration, the samples were embedded in 100% resin. Ultra-thin sections were placed on copper grids, stained with 2% uranyl acetate and lead citrate, and examined using a transmission electron microscope (TEM JEM 1200 Exll, Jeol, USA). The integrity of cytoplasmic organelles and cytoplasmic and nuclear membranes of PG oocytes was investigated.

### Biochemical analysis

Ovarian tissue fragments were homogenized in ice-cold (0 °C to 4 °C) 50 mMTris-HCl, pH 7.4 (1/10, w/v). The homogenate fragments were centrifuged for 10 min at 3000 × g.Subsequently, the pellet was discarded, and the obtained low-speed supernatant (S1) was used to determine the reactive species andantioxidant capacity and mitochondrial activity.

The levels of reactive oxygen species (ROS) were determined by a spectrofluorimetric method^18^ using the dichlorodihydrofluoresceindiacetate (DCHF-DA) assay. The samples (20 µL) were incubated with DCHF-DA (1 mM), and the oxidation of DCHF-DA to fluorescent dichlorofluorescein was measured using the fluorescence intensity at 520 nm emission (with 480 nm excitation) at 120 min after the addition of DCHF-DA to the medium. Each sample was analyzed in triplicate.

The total antioxidant capacity was determined by the “ferric reducing antioxidant potential” (FRAP), where the antioxidants present on samples were evaluated as reducers of Fe^+3^ to Fe^+2^, which is chelated by 2,4,6-Tri(2-pyridyl)-s-triazine (TPTZ), forming the complex Fe + 2-TPTZ, with max absorption at 593 nm^19^. An ascorbic acid standard curve was realized, and the results were expressed in μg equivalent of ascorbic acid.Each sample was analyzed in triplicate.

### Mitochondrial activity

Mitochondrial activity was assessed by thiazolyl blue tetrazolium bromide (MTT) staining using spectrophotometry^20^. The principle of this method is based on the reduction of MTT to formazan crystals by the mitochondrial succinyl dehydrogenase enzyme, active only in living cells. Immediately after warming, ovarian tissue fragments were homogenized in phosphate-buffered saline (PBS) and incubated with MTT (5 mg/mL) for 120 min at 25 °C. The supernatant (200 μL) was carefully removed and dimethyl sulfoxide (200 μL) was added to dissolve the formed crystals. Next, 200 μL of the colored solution were transferred for absorbance analysis at 570 nm on the SpectraMax® 250 Microplate Spectrophotometer. The greater the color intensity was, the higher the mitochondrial activity. Each sample was analyzed in triplicate.

### DNA damage

The comet assay was performed under alkaline conditions, according to the procedure of Singh *et al*.^21^, Collins^22^ and adaptations from Da Silva *et al*.^23^. Oocytes were isolated by density centrifugation (30 min, 18 °C, 400 × g) on Ficoll-Paque™ Plus. To examine basal DNA damage, aliquots (20 μL) of the oocytes were immediately collected after centrifugation, mixed with 90 *μ*L of low melting point agarose (0.7% in phosphate buffer) and added to microscope slides pre coated with 1.5% agarose. The slides were then incubated in ice-cold lysis solution (2.5 M NaCl, 100 mM EDTA, 10 mMTris, 20mMNaOH, pH 10.2, 1% Triton X-100, and 10% DMSO). For the DNA repair study, the cells were treated with H_2_O_2_ (10 μM, 5 min on ice) or with methyl methane sulfonate (MMS) (8 × 10− 5 M, 1 h at 37 °C).

After treatment, the cells were washed with PBS (centrifugation, 5 min, 4 °C, 200 × g) and incubated in RMPI 1640 (200 μL) with 10% fetal calf serum. Aliquots of the suspension (20 μL) were immediately collected or after 60 and 180 min of post-incubation, mixed with (80 μL) 0.75% low melting point agarose and cast onto microscope slides pre coated with 1.5% normal agarose. After 24 h at 4 °C, the slides were removed from the lysis solution and placed in an electrophoresis unit filled with fresh electrophoresis buffer at 4 °C. In the alkaline version of the comet assay (10 M NaOH,1 mM EDTA, pH > 13), 20 min of denaturation and 15 min of electrophoresis were used. For the neutral version of the comet assay (3 M sodium acetate, 1 M Tris, and pH = 8.5), 1 h of denaturation and 1 h of electrophoresis were used. In both versions of the comet assay, after electrophoresis, the slides were neutralized (0.4 M Tris, pH 7.5) and washed in water. The slides were dried overnight at room temperature, then fixed and stained with 20 μLof 4,6-diamidino-2-phenylindole. Each slide was viewed by fluorescence microscopy, and the degree of damage was visually scored. A total of 100 comets on each slide were assigned a score from 0 to 4, depending on the fraction of DNA extracted from tail. The overall score for each slide was between 0 (undamaged) and 400 (completely damaged).

### Statistical analyses

Normality (Kolmogorov-Smirnov) and homogeneity (Levene’s test) were previously verified. Variables ROS showed non-normal distribution; therefore, all data were log-transformed to show a normal distribution prior to analysis. Statistical analysis was performed by using one-way analysis of variance (ANOVA). Tukey’s test, at 95%, was used to assess significant differences among the means. Student’s t test was applied for biochemical analysis data and mitochondrial activity. The results of the comet assay did not show normal distribution, even after data transformation; thus, a non-parametric Kruskal-Wallis analysis was applied, followed by Dunn’s test.

## Results

### Experiment 1

**Figure 1:**
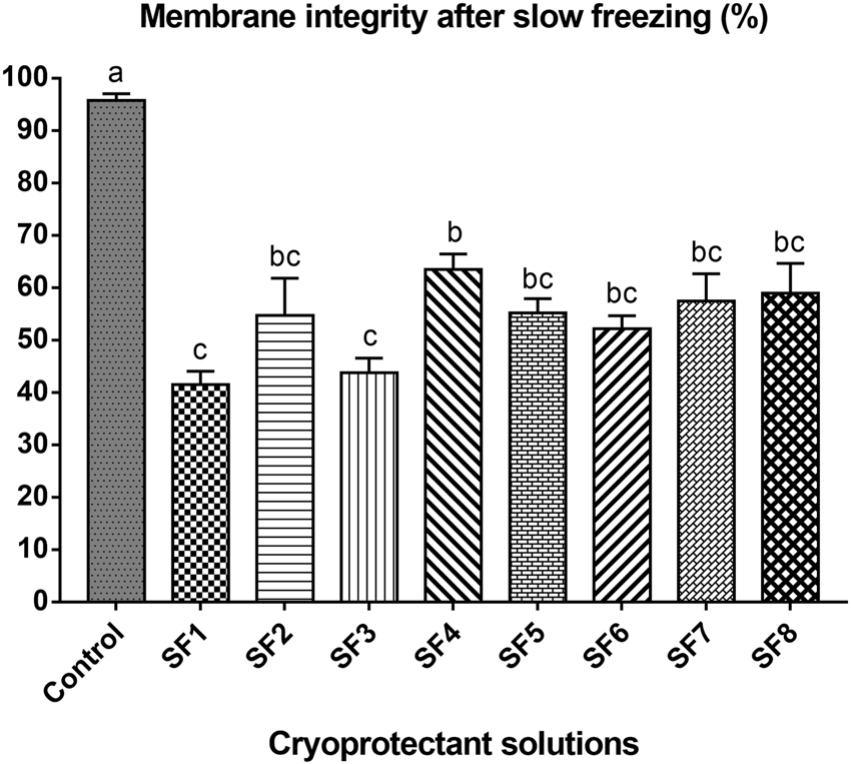
Membrane integrity after vitrification. V1 (1.5 M methanol + 5.5 M Me_2_SO + 0.5 M sucrose); V2 (1.5 M methanol + 5.5 M Me_2_SO + 0.5 M sucrose + 10% egg yolk solution); V3 (1.5 M methanol + 5.5 M Me_2_SO + 0.5 M trehalose); SV4 (1.5 M methanol + 5.5 M Me_2_SO + 0.5 M trehalose + 10% egg yolk solution). Control = fresh ovarian tissue fragments. Mean ± SE followed by different letters differ by Tukey’s test (P = 0.0004).

The membrane integrity of PG oocytes in the control group (fresh ovarian tissue fragment) was significantly higher compared to all vitrification solutions; however, there was no significant difference among the treatments (Fig. 1).

**Figure 2:**
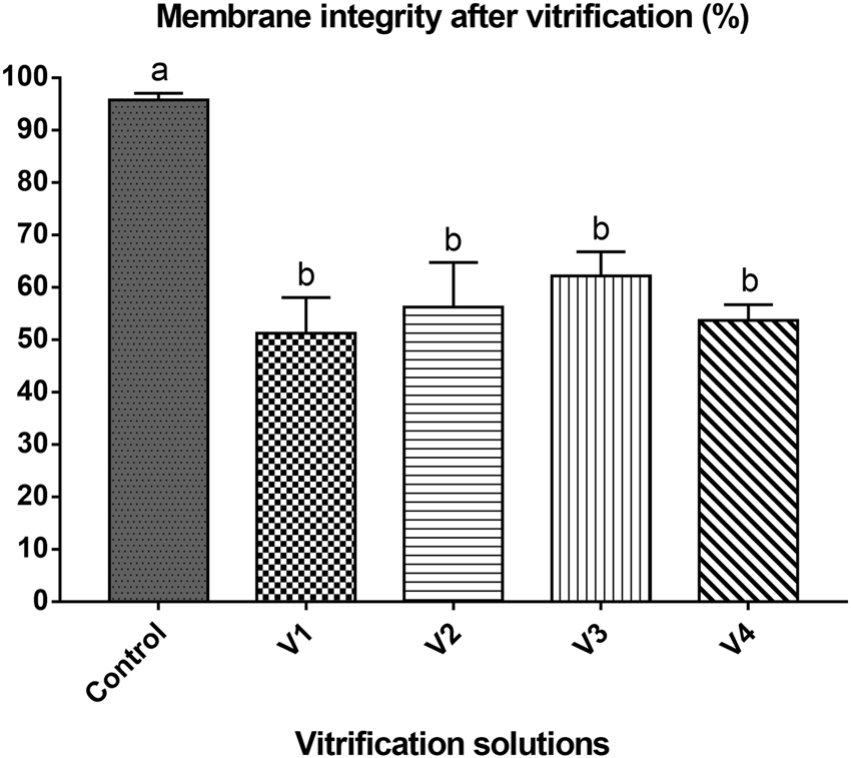
Membrane integrity after slow freezing. SF1 (2 M methanol + 0.1 M sucrose); SF2 (2 M methanol + 0.1 M sucrose + 10% egg yolk solution); SF3 (2 M methanol + 0.1 M trehalose); SF4 (2 M methanol + 0.1 M trehalose + 10% egg yolk solution); SF5 (2 M Me_2_SO + 0.1 M sucrose); SF6 (2 M Me_2_SO + 0.1 M sucrose + 10% egg yolk solution); SF7 (2 M Me_2_SO + 0.1 M trehalose); SF8 (2 M Me_2_SO + 0.1 M trehalose + 10% egg yolk solution). Control = fresh ovarian tissue fragments. Means ± SE followed by different letters differ by Tukey’s test (P < 0.0001).

The membrane integrity after slow freezing with the eight cryoprotectant solutions is shown in Fig. 2.There was a significant difference in membrane integrity between the control group and the slow freezing treatments (Fig. 2). PG oocytes showed the best percentage of membrane integrity (63.5 ± 2.99%) when SF4 solution was employed. When ovarian tissue fragments were frozen in SF1 and SF3, the membrane integrity decreased to 41.5 ± 21.0% and 43.75 (P < 0.05),respectively. Based on these results, SF4 solutionwas used for the subsequent experiments.As there was no significant difference among vitrification solutions, the solution containing egg yolk that presented the highest membrane integrity percentage (V2) was selected for Experiment 2.

### Experiment 2

**Figure 3:**
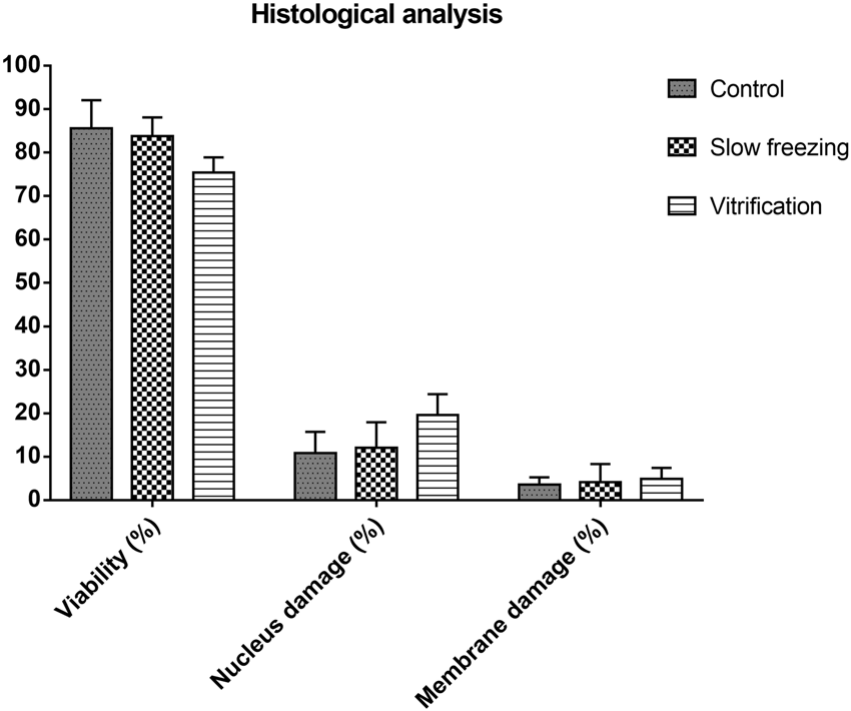
Histological analysis after slow freezing and vitrification. Not significant (P > 0.05). Cell integrity (%): P = 0.3452; Nuclear damage (%): P = 0.4627; Membrane damage (%): P = 0.9517.

**Figure 4:**
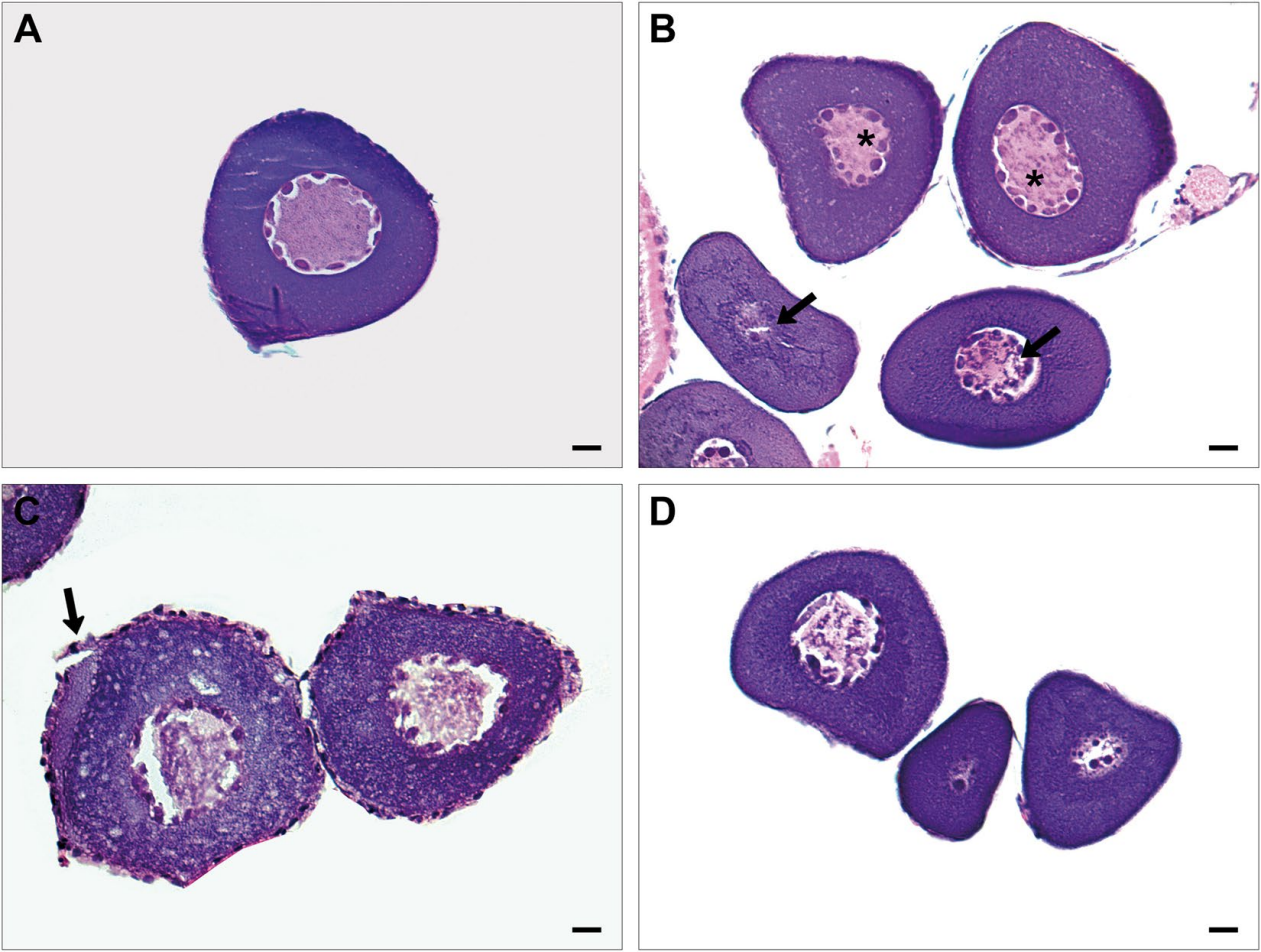
Histology of fresh (**A**), vitrified (**B**) and frozen (**C**,**D**) primary growth (PG) oocytes of zebrafish (*Danio rerio*) after thawing.In A, fresh PG oocyte with intact nucleus and cell membrane.In B,vitrified PG oocytes with condensed chromatin (arrow) and oocytes with intact nucleus (asterisk).In C, frozen PG oocyte with follicular membrane rupture (arrow). In D, three frozen PG oocytes with nucleus damage.Light microscope 40x. Stain: HE. Bar = 10 µm.

There was no significant difference (P > 0.05) among control, slow freezing and vitrification in the histological analysis (Fig. 3). In the evaluation of nuclear damage (Fig. 4), vitrification showed an increase (19.64%) in relation to freezing (12.04%); however, this difference was not statistically significant (P = 0.4627).

**Figure 5:**
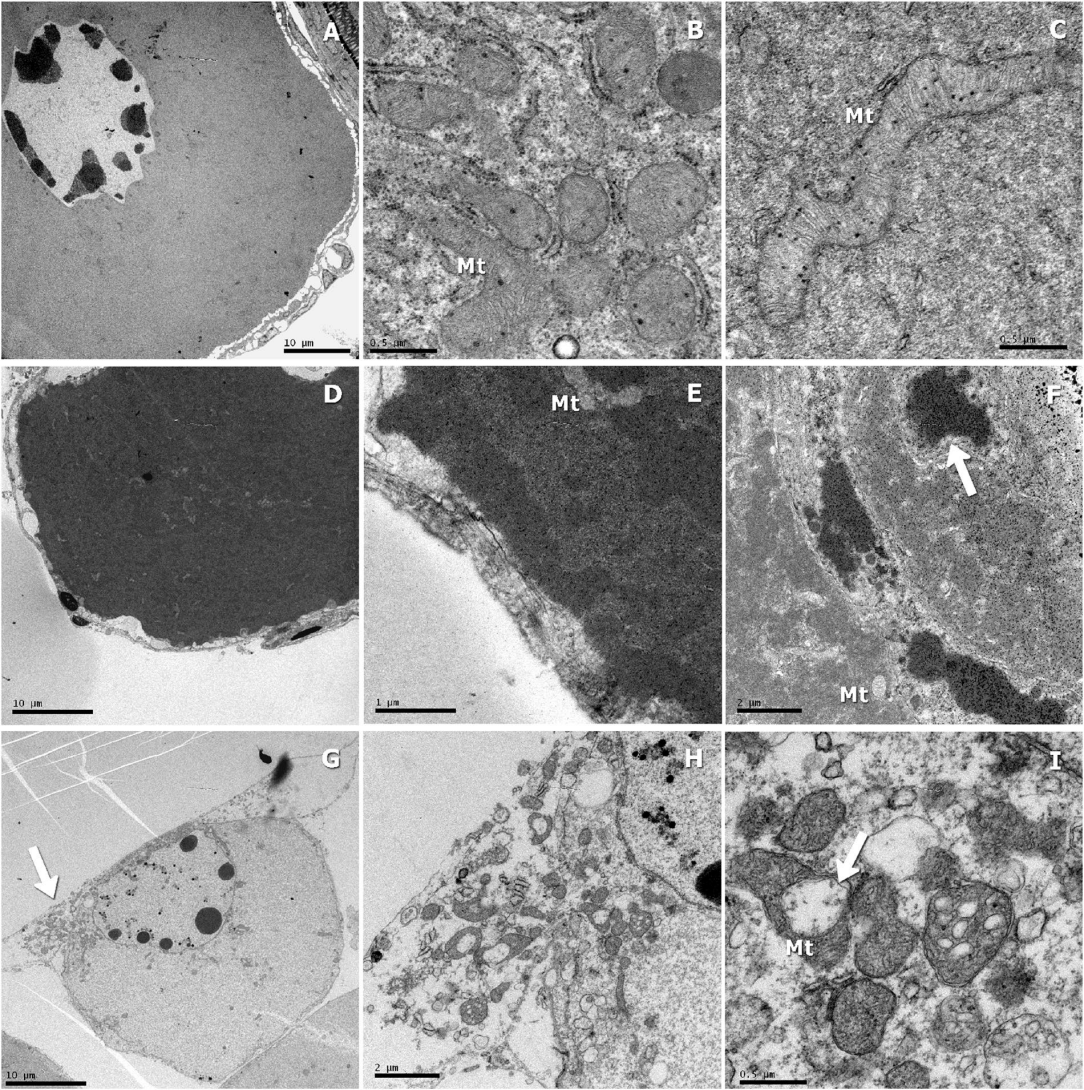
Transmission electron microscopy (TEM) of fresh (control), vitrified and frozen primary growth (PG) oocytes of zebrafish (*Danio rerio*). (**A**–**C**) Fresh PG oocytes with normal cell organelles and intact plasma and nuclear membranes. (**B**,**C**) Presence of large number of mitochondria in the cytoplasm. (**D**–**F**) Vitrified PG oocyte showing an intact plasma membrane, chromatin condensation (arrow), signs of a ruptured nuclear membrane and a lower number of mitochondria. (**G**,**H**) Frozen PG oocyte showing a ruptured plasma membrane and a loss of intracellular contents (arrow). (**I**) Cytoplasm of a frozen PG oocyte with swollen mitochondria (arrow). PG = primary growth, Mt = mitochondria.

Figure 5 shows morphological ultrastructural features of fresh, vitrified and frozen PG oocytes by TEM.The ultrastructural observations of fresh PG oocytes showed a normal aspect of the organelles and intact plasma and nuclear membranes (Fig. 5). In addition, fresh PG oocytes had a large number of mitochondria in the cytoplasm (Fig. 5B). Although the vitrified PG oocytes hadintact plasma membranes (Fig. 5D), there was nuclear condensation (pyknosis) and signs of a ruptured nuclear membrane (Fig. 5F). The TEM also showed a lower number of mitochondria in the cytoplasm of vitrified PG oocytes (Fig. 5E). The ultrastructural observations of frozen PG oocytes showed the rupture ofthe plasma membrane andthe loss of intracellular contents (Fig. 5G,H). In addition, a large number of damaged mitochondria were observed in frozen PG oocytes (Fig. 5I).

**Figure 6:**
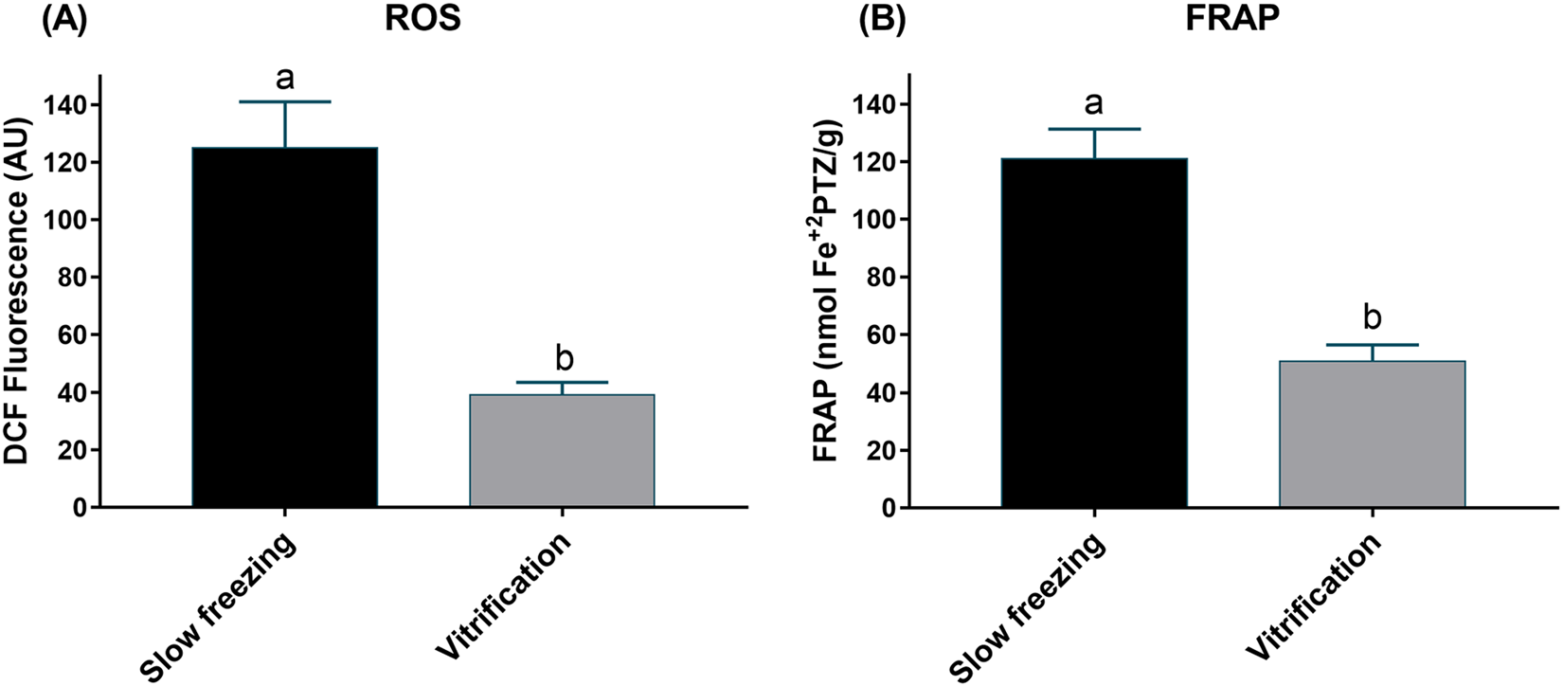
(**A**) Reactive oxygen species (ROS) by DCFH, and (**B**) total antioxidant capacity by FRAP (Ferric reducing/antioxidant power) after slow freezing and vitrification. Means ± SE followed by different letters differ by Student’s t test. ROS: P < 0.0001; FRAP: P < 0.0001.

Frozen ovarian tissue showed higher ROS levels than vitrified ovarian tissue (P < 0.0001; Fig. 6A). Compared to frozen ovarian tissue, vitrified ovarian tissue showed a reduced antioxidant capacity as measured by FRAP (P < 0.0001; Fig. 6B).

**Figure 7:**
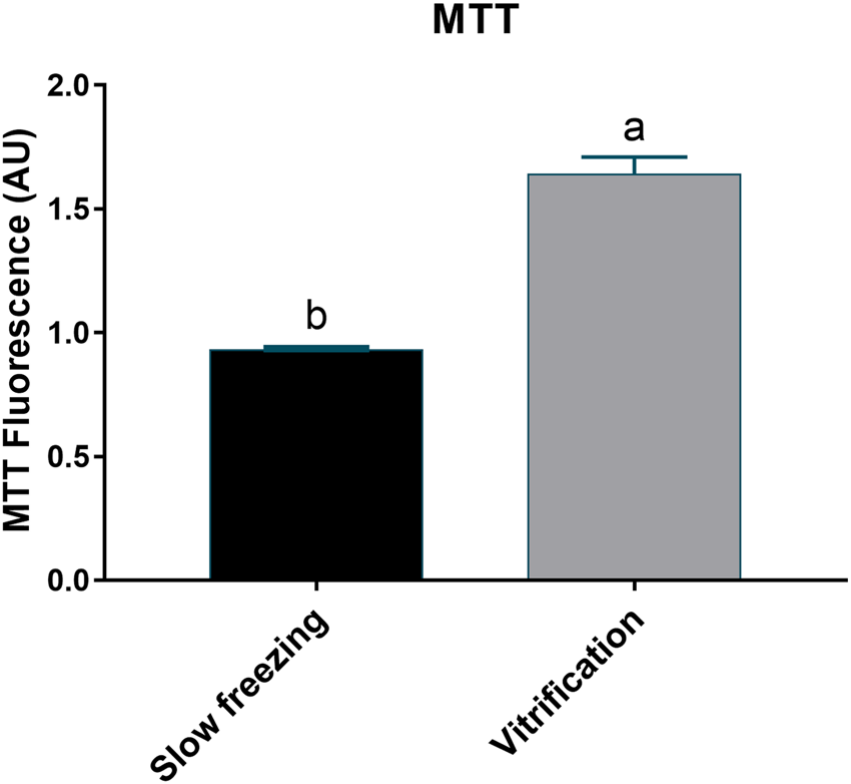
Mitochondrial activity by thiazolyl blue tetrazolium bromide (MTT). Means ± SE followed by different letters differ by Student’s t test (P = 0.0081).

The mitochondrial activity in vitrified ovarian tissue was significantly higher when compared tothat in frozen ovarian tissue (P = 0.0081; Fig. 7).

**Figure 8:**
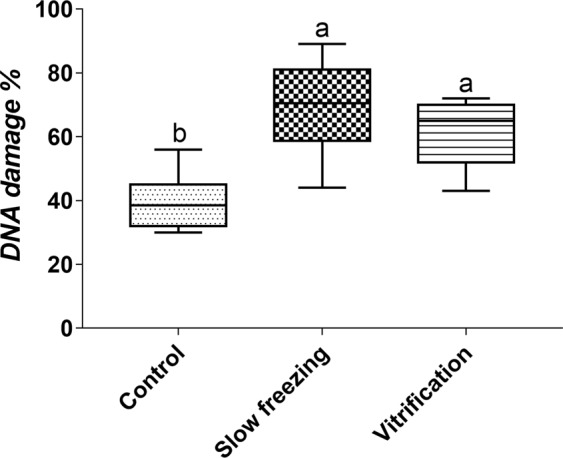
DNA damage (%) by comet assay of fresh (control), frozen and vitrified primary growth (PG) oocytes of zebrafish (*Danio rerio*). Means ± SE followed by different letters differ by the analysis of Kruskal-Wallis, followed by Dunn’s test (P = 0.0013).

Cometa assays showed no difference between vitrification (61.86%) and slow freezing (69.63%; P > 0.05; Fig. 8). However, slow freezing showed a more significant difference in relation to the control (39.6%; P = 0.0013), whereas vitrification showed the least significant P in relation to the control (39.6%; P = 0.0382).

## Discussion

Cryopreservation promotes changes in the physical and chemical properties of oocytes, including cell membrane damage, increased production of reactive oxygen species, DNA damage, and mitochondrial depolarization^24–28^. Therefore, cell morphology, oxidative stress, DNA damage,mitochondrial activity and structure were measure after slow freezing and vitrification.

Part of the damage caused by the cryopreservation of fish oocytes occurs at the plasma membrane. Therefore, the development of more efficient cryopreservation methods depends on the use of substances that protect the integrity of the oocyte membrane during cooling. Previous studies have reported that the addition of egg yolk in cryoprotectant solution had a protective effect on the integrity of both mouse oocytes and cumulus cells^17^. In a study with the frozen testes of rainbow trout, type A spermatogonia (ASGs) obtained from testes whose cryoprotectant solution contained 10% (vol/vol) egg yolk displayed the highest rates of survival^16^. The present study provides the first evidence of the effect of egg yolk on oocyte membrane integrity after vitrification and slow freezing of fish ovarian tissue. Although the egg yolk did not show a positive effect after vitrification, when added to freezing solution containing 2 M methanol and 0.1 M trehalose, the membrane integrity after slow freezing increased to 63.5%. The major concern during the slow cooling of cells is the potential formation of intracellular ice that may cause cell and membrane damage and directly affect viability^29^. The action of egg yolk may be attributed tophospholipids^30^, cholesterol^31^ and low-density lipoprotein^32^ contents, which are incorporated in the cell membrane, providing protection to the oocyte membrane against osmotic shock and cryoinjuries during slow freezing^33,34^.

Compared to slow freezing, vitrification is able to prevent the formation of intracellular and extracellular ice crystals, reducing the risk of mechanical damage to cells. However, morphological evaluation by histology showed no difference between the slow freezing and vitrification techniques, as well as no difference between fresh and cryopreserved PG oocytes in membrane and nuclear damage. The cryopreservation of immature oocytes may reduce membrane damage because PG oocytes are surround by a single layer of squamous follicle cells that lie on a distinct basement membrane. While the cortical alveolar stage presentsthree layers, the newly formed inner layer is thickest and more architecturally complex^35^.

Several stress factors associated with cryopreservation are known as initiators of apoptotic cell death. Further, the combination of cell physical damage with cell stresses experienced during the freeze-thaw procedure can result in necrosis^36^. Apoptosis describes a specific morphological aspect of cell death and is accompanied by pyknosis (reduction of cellular volume and chromatin condensation), nuclear fragmentation (karyorrhexis), and little or no ultrastructural modifications of cytoplasmic organelles^37^. In the present study, these morphological features were observed in the ultrastructural analysis of vitrified PG oocyte, indicating the occurrence of cellular apoptosis during vitrification. The ultrastructural observations of frozen PG oocytes showed organelle swelling, plasma membrane rupture and intracellular content loss, which are characteristic of cellular necrotic cell death. In this sense, vitrification was more able to morphologically conserve the follicular structures than slow freezing.

The number and distribution of mitochondria and energy (ATP) production are critical factors that influence the oocyte maturation, fertilization, and embryo development^38^. Zampolla *et al*.^38^ showed that higher concentrations of methanol (3 and 4 M) induced a decrease in mitochondrial membrane potential and the loss of mitochondrial distributional arrangement in zebrafish ovarian follicles at stage III, compromising mitochondrial function. In the present study, the ultrastructural analysis of fresh PG oocytes showed a large number of mitochondria in the cytoplasm. Despite the smaller number of mitochondria in the cytoplasm of vitrified PG oocytes, the structure of these mitochondria appeared well preserved. In contrast, in frozen PG oocytes showed a large number of damaged mitochondria. For this reason, mitochondrial activity in vitrified ovarian tissue was higher when compared to that in frozen ovarian tissue. In addition, frozen ovarian tissue showed higher ROS levels and total antioxidant capacity than vitrified ovarian tissue. These results can be explained by the mitochondrial damage observed in frozen PG oocytes. Mitochondrial antioxidant enzymes detoxify ROS; however, excess ROS generation may overwhelm the capacity of these defenses, leading to mitochondrial damage^39^.

The comet assay is a simple method for measuring deoxyribonucleic acid (DNA) strand breaks in individual cells^22^, and this assay is widely used to measure DNA damage in reproductive cells^40–46^. Freezing and thawing increase DNA strand breaks, and previous studies have associated this increased damage with the oxidative stress that occurs during cryopreservation^47^. In addition, studies showed that vitrification was associated with significantly less DNA fragmentation in human and murine primordial follicles compared with that in slow freezing^46,48^. However, in the present study, there was no difference between vitrification and slow freezing techniques on the cryopreservation of zebrafish ovarian tissue containing immature follicles.

Vitrification, besides avoiding crystallization, is a relatively simple, fast and inexpensive method of cryopreserving ovarian tissue when compared to slow freezing. Thus, vitrification is a useful tool for germplasm preservation in case of unexpected death of animals of great genetic value.

## Conclusion

Mitochondria are essential organelles involved in many cellular functions. The mitochondrial damage observed by ultrastructural analysis and the lower mitochondrial activity could be responsible of the increment in the levels of ROS observed in frozen oocytes. Therefore, the vitrification technique was more efficient than slow freezing in preventing mitochondrial damage and avoid oxidative stress on cryopreservation of zebrafish ovarian tissue containing immature follicles. In addition, lower ROS production and better preserved PG oocytes may improve tissue function during *in vitro* maturation.
